# Training the dairy workforce: Delivery methods, evaluation, and outcomes across worker tasks—A mini-review

**DOI:** 10.3168/jdsc.2026-1120

**Published:** 2026-09-18

**Authors:** W. Heuwieser, A. Zapata Salazar

**Affiliations:** Department of Population Medicine and Diagnostic Sciences, College of Veterinary Medicine, Cornell University, Ithaca, NY 14853

## Abstract

As dairy farms consolidate and rely increasingly on hired and immigrant labor, consistent best-practice routines depend on how effectively a diverse workforce can be trained. This review examines training for the tasks dairy workers perform, such as milking, udder health care, calf care, antimicrobial stewardship, euthanasia, and occupational safety, as well as the methods used to deliver training. Training, whether through workshops, hands-on demonstrations, online courses, or standard operating procedures, reliably increases knowledge and confidence, with the most pronounced improvements found for tasks performed rarely or not previously taught. Hands-on instruction favors technical skill acquisition, whereas online and SOP-based approaches provide flexibility, repeatability, and easy translation for workers constrained by long shifts and turnover. Persistent challenges include language and literacy barriers and the dominance of reaction- and learning-level evaluation. Few studies have documented behavior change or herd-level effects. More robust study designs and the extension of evaluation toward impact are critical.

## Graphical Abstract

**Figure:**
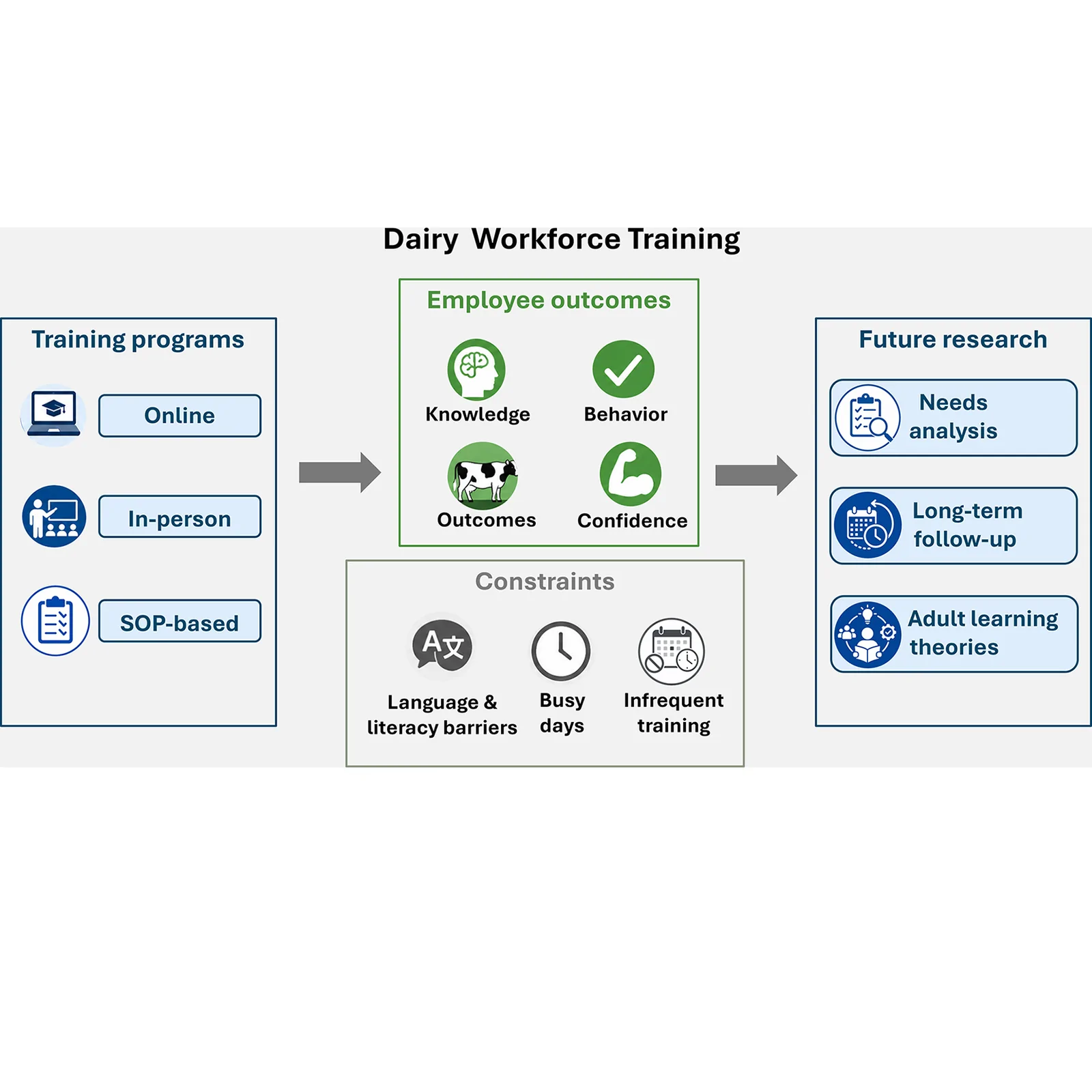

The performance and well-being of dairy employees influence animal health and welfare, milk quality, and food safety (Barkema et al., 2015; Erskine et al., 2015; Neukirchner et al., 2024). Although on-farm technologies such as sensors and machine-vision systems are expanding into dairy operations, the successful implementation of best management practices continues to depend on employees and the consistency with which they perform tasks (Durst et al., 2018). In the United States, most frontline dairy work is performed by Latino employees, many of whom enter with limited agricultural experience and face language and cultural barriers that can complicate supervision, communication, and training (Erskine et al., 2015; Rovai et al., 2016; Durst et al., 2018; Alanis et al., 2022; Rodriguez et al., 2025).

Evidence indicates that structured training programs improve employee knowledge, behavior, and on-farm performance, while also strengthening teamwork, communication, and job satisfaction (Rovai et al., 2016; Sischo et al., 2019; Moore et al., 2020). In the United States, a variety of extension education programs are available. Furthermore, the National Dairy FARM Animal Care Program requires annual continuing education for employees performing specific animal care tasks. Nonetheless, training practices vary considerably across the industry (Martins et al., 2019). High employee turnover, informal or unstructured training, language barriers, and protocol drift contribute to variation in task execution and poor protocol compliance (Erskine et al., 2015; Alanis et al., 2022; Stup et al., 2025).

The consequences of inconsistent training are evident across multiple areas of dairy production, such as calf care, udder health care, worker safety, and animal welfare. Inadequate training or poor adherence to protocols has been associated with variation in milk quality (Rodriguez et al., 2025), inadequate milking equipment checks (Alanis et al., 2022), lower attitude scores toward antimicrobial stewardship (Rovai et al., 2016), and poorer safety practices (Vázquez et al., 2025). Together, these shortcomings show the need for systematic training to reduce mastitis risk factors.

This review summarizes the recent evidence regarding employee training in the dairy industry, highlights gaps in implementation and assessment, and explores future approaches informed by instructional design models relevant to workplace learning in adult education. Specifically, we aim to (1) review studies from the last 10 years on dairy farm employee training, (2) assess whether these studies are based on or discuss principles relevant to adult education, and (3) identify opportunities for future research.

To identify studies focusing on training dairy employees, we conducted literature searches in PubMed (https://pubmed.ncbi.nlm.nih.gov) and Google Scholar (https://scholar.google.com) on September 14, 2025, and again on April 30, 2026. The searches used combinations of terms related to dairy employees, milker, personnel, workforce training, learning, education, standard operating procedures (**SOP**), safety, stewardship, and employee management. We limited the search to the past 10 years to focus on training delivered under the technological and demographic conditions that characterize the current dairy industry (e.g., an increasingly Latino workforce, widespread e-learning adoption).

Only studies designed around a discrete training intervention and describing a training outcome were included. Studies that targeted veterinary students, farm managers, or supervisors were not considered. Conference proceedings, reviews, abstracts, cross-sectional surveys, interviews, and focus group discussions were also removed. In a second step, the reference lists of eligible articles were screened for further studies.

A total of 29 papers were identified (listed in supplemental material; see Notes), and 15 studies met the inclusion criteria and evaluated training interventions for dairy farm employees (Table 1). Training topics included milking procedures, udder health, calf care, antimicrobial stewardship, euthanasia decision-making, occupational safety, and milk quality management. Studies were conducted either in person (n = 6) or online (n = 6). One study combined 6 pre-recorded modules with live sessions. Most studies relied on convenience samples of farms from specific regions, with a mean ± SD of 24.5 ± 17.7 farms and 293.4 ± 425.6 employees enrolled (n = 11). Study details are summarized in Table 1. Only 2 of the 13 reviewed studies reported a formal sample size calculation for the training component (Merenda et al., 2023; Rodriguez et al., 2025). Although Portillo-González et al. (2024) provided sample size information, it pertained to the antimicrobial use (**AMU**) quantification component rather than the training component per se. The remaining 10 studies did not report an a priori sample size calculation. Similarly, none of the reviewed publications reported a formal training needs assessment before the development or implementation of the intervention.

**Table 1:** Summary of 15 studies on dairy employee training considering format, topic, and findings^1^

Author, year	Country (state or province)	Farms (n)/ employees (n)	Training topic	Training format	Language (evaluation)	Results
Alanis et al. (2022)	United States (NY)	15/95	Milking equipment checks, malfunction detection	Online	ENG, SPA (post-test)	95% reported confidence in equipment checks post-training; 86% more confident reporting problems to managers
Garzon et al. (2023)	United States (CA, OH)	12/25	Antimicrobial stewardship	Combined (online + hands-on session)	ENG, SPA (pre/post-test)	32% average knowledge gain; significant attitude improvement in 7 of 13 stewardship-related questions; farm size associated with greater knowledge gain
Hesse et al. (2019)	Germany	35/117	Colostrum management	Online	GER (post-test)	78% felt more confident in task performance; 80% convinced to work more accurately
Heuwieser et al. (2024)	United States (NY)	15/60	Udder health, dry-off treatments	Online (SOP-based)	ENG, SPA (pre/post-test, 2–3 wk)	OSCE scores rose significantly (*P* < 0.001) for 2 modules; 83% and 90% of participants improved their behavior
Juárez-Carrillo et al. (2017)	United States (WI)	67/836	Occupational safety and health	In-person	ENG, SPA (pre/post-test)	25% knowledge gain (*P* < 0.01); 67% of workers had never received prior safety training; workers expressed confidence and intention to adopt safe practices
Merenda et al. (2023)	United States (NC, CA, CT)	30/81	Euthanasia decision-making	Online	ENG, SPA (pre/post-test)	Significant improvement in perceived euthanasia knowledge; younger and less experienced caretakers benefited most
Meyerhoff et al. (2016)	United States (NY)	NR/35	First aid	In-person	ENG, SPA (pre/post-test)	Significant improvement in first aid knowledge
Neukirchner et al. (2024)	Germany	NR/301	Calf care (8 tasks)	Online (SOP-based)	GER (pre/post-test)	Wish for SOP (69%) exceeded existing SOP (13%); 85% agreed SOP suited for training; confidence in tasks increased
Neukirchner et al. (2024)	Germany	NR/301	Calf care (8 tasks)	Online (SOP-based)	GER (pre/post-test)	High self-perceived learning effects (49%–80%); 90% rated courses easy to use, 97% understandable; interest declined over time
Portillo-González et al. (2024)	United States (OH, CA)	18/25	Antimicrobial stewardship	Combined (online + hands-on session)	ENG, SPA (post-AMU assessment)	No significant change in AMU in trained vs. control farms; 32% knowledge gain in trained workers
Rodriguez et al. (2018)	United States (NM, TX, CO, KS, NY)	40/1,432	Occupational safety and health	Online	ENG, SPA (pre/post-test)	Significant knowledge gain (74.2% to 92.5%); 95% applied safety techniques 3 mo post-training; 100% reported performing job more safely
Rodriguez et al. (2025)	United States (MI, OH)	16/112	Milking routine compliance, mastitis identification, milk quality	In-person	ENG, SPA (pre/post-test)	Knowledge increased from 49% to 68%; disinfectant contact time increased by 9 s; mastitis detection increased immediately after training
Rovai et al. (2016)	United States (SD)	3/75	Animal care, milking procedures, mastitis, worker safety	In-person	SPA (post-test)	85% of workers reported greater job confidence; owners noted improved employee behavior and hygiene awareness; SCC increase attributed to seasonal effects, not training
Vázquez et al. (2025)	United States (MN, SD)	19/360	Occupational health and safety	In-person	SPA (pre/post-test)	Significant knowledge gains after all lessons (*P* < 0.001); managers reported improved awareness, positive behavior, and fewer incidents
Winder et al. (2018)	Canada (ON)	NR/54	Disbudding in calves	Online vs. hands-on vs. combination	ENG (online vs. hands-on comparison)	Similar ability to provide an effective cornual nerve block for online and hands-on training; a combined approach increased confidence

1 NR = not reported (participants were recruited via self-registration; individual farm count was not tracked); ENG = English; SPA = Spanish; GER = German; OSCE = objective structured clinical examination; AMU = antimicrobial use; SOP = standard operating procedures.

Only 5 papers explicitly named at least 1 training framework. The Kirkpatrick model was cited in 2 manuscripts (Rodriguez et al., 2018; Heuwieser et al., 2024); Bloom's taxonomy in 2 (Rodriguez et al., 2018; Garzon et al., 2023); and Knowles' andragogy in 2 (Juárez-Carrillo et al., 2017; Garzon et al., 2023). However, all manuscripts incorporated elements consistent with adult learning principles to some degree, such as bilingual delivery, rationale-first content, hands-on components, active training, spaced training, and small-group formats, often without providing a definition or citation.

The manuscripts therefore varied not only in whether they explicitly identified educational frameworks, but also in how training content was delivered, with online and in-person instruction representing the most common approaches. Online learning offers a practical way to address the logistical constraints of in-person training on dairy farms (Alanis et al., 2022). Its effectiveness appears greatest when programs are bilingual, visually rich, and text-limited to accommodate different literacy levels among employees (Alanis et al., 2022; Merenda et al., 2023). Across dairy settings, online and in-person training has been associated with gains in knowledge, confidence, accuracy, and observed behavior. Online training can also provide greater flexibility by allowing employees on different shifts and at different locations to complete training at convenient times. In some cases, training materials can also be accessed offline after being downloaded (Rodriguez et al., 2018; Hesse et al., 2019; Alanis et al., 2022; Heuwieser et al., 2024). Successful implementation, however, depends on more than the availability of digital content. Management support, dedicated training time, and the development of a workplace learning culture appear essential for course completion and employee engagement (Alanis et al., 2022) such as active participation and interaction with online learning materials.

In-person training may be particularly well suited for hands-on practice, development of psychomotor skills that integrate cognitive processes with physical movement, and topics that require interaction or farm-specific discussion. Studies using participatory and active learning approaches have reported improvements in knowledge, compliance with milking routines, herd-level outcomes, and safety-related intentions (Juárez-Carrillo et al., 2017; Rodriguez et al., 2025). Evidence directly comparing online and in-person training in dairy contexts remains limited. To our knowledge, only 1 Canadian study evaluated these approaches for teaching a complex psychomotor skill, specifically cautery disbudding technique, although the target audience was dairy producers rather than employees. In that study, online modules were as effective as hands-on training for the successful performance of a cornual nerve block for calf disbudding, whereas hands-on training resulted in greater participant self-confidence (Winder et al., 2018).

The strengths of each training format also appear to shape its main limitations. Online-only training limits opportunities for hands-on practice and results in lower technical confidence, whereas in-person training is more labor-intensive, less scalable, and more vulnerable to disruption from farm schedules (Rodriguez et al., 2018; Alanis et al., 2022). A blended approach that combines in-person and online training, potentially enhanced by emerging technologies such as artificial intelligence and virtual reality, may therefore offer additional value, although this remains to be established empirically.

Beyond the mode of training delivery, several studies evaluated training programs supported by SOP. Findings suggest that SOP-based training is valuable not simply because it provides information, but because it helps standardize how critical tasks are performed across employees, shifts, and farms. Standard operating procedures and checklists are designed to reduce human error and improve process and product quality by translating best practices into consistent, repeatable actions (Barkema et al., 2015; Barragan et al., 2016).

This review provides evidence that online training based on 3 ready-made SOP improved both confidence and perceived accuracy in task execution (Hesse et al., 2019). In a larger study featuring a series of 8 SOP-based e-learning courses focused on calf care, participants reported increased confidence across topics, particularly for infrequently performed tasks such as tube feeding, emergency care, and colostrum quality testing. Most participants (85%) agreed that SOP are suitable for training new employees, indicating strong perceived value for onboarding and skill reinforcement (Neukirchner et al., 2024). Similarly, an interactive e-learning course provided ready-made SOP related to udder health across 5 modules delivered in Spanish and English. Participants completed the modules independently or via narrated videos. Milker behavior was assessed pre-training and 2 to 3 weeks post-training using an objective structured clinical examination (**OSCE**) for dry-cow treatment and internal teat sealant administration. Average scores for correct implementations increased significantly for both modules from pre- to post-training, indicating behavior change (Heuwieser et al., 2024).

These benefits seem particularly relevant to dairy operations because online delivery can reduce scheduling and geographic barriers while supporting bilingual, visually oriented instruction for a diverse workforce (Alanis et al., 2022; Merenda et al., 2023). However, the literature also suggests that SOP are unlikely to be effective when used as static documents alone. Their value appears to depend on whether they are embedded in active training, reinforcement, and accountability structures. Some evidence from a Danish survey associated SOP-based training programs with more favorable udder health outcomes than SOP distribution without structured training (Farre et al., 2024).

Despite these benefits, adoption of written SOP on dairy farms remains limited (Espadamala et al., 2016; Beecher et al., 2025), even though employees expressed interest in having structured guidance (Neukirchner et al., 2024). This contrast suggests that the main challenge is no longer whether SOP are conceptually useful, but how to implement them in ways that are practical, accessible, and integrated into daily workforce management.

The reviewed studies also reported participants' demographics and work experience. Although demographic data were often limited and not systematically collected to evaluate associations with or influence on training effectiveness or herd-level outcomes, they still provide a useful snapshot of the learner populations and evidence of the challenges of training a diverse workforce, including limited prior dairy knowledge, time constraints, high turnover, and linguistic diversity (Alanis et al., 2022; Rodriguez et al., 2025). Moreover, this information could be used to improve the design, readability, and delivery of future training efforts by using less text-heavy e-learning modules (Meyerhoff et al., 2016), increasing the use of visual and audio materials, and allocating dedicated training time to address these barriers (Alanis et al., 2022; Heuwieser et al., 2024).

Many employees lacked formal agricultural training before entering the dairy industry. In one study, 46% of milkers had no prior experience milking cows before starting at their current farm (Alanis et al., 2022). Educational attainment varied across studies. In 2 US studies evaluating milking procedures and occupational safety, 47.1% and 58.7% of participants, respectively, had not completed high school (Rodriguez et al., 2025), compared with 17.6% in a German calf care study. Participants' literacy was not assessed, even though some evidence suggests that reading and writing abilities may be limited (Meyerhoff et al., 2016; Juárez-Carrillo et al., 2017). Language barriers were also common. Several studies used English- and Spanish-language training materials (Alanis et al., 2022; Portillo-González et al., 2024; Rodriguez et al., 2025), and 22.4% of workers in one large US study reported speaking an Indigenous language, such as K'iche', rather than English or Spanish, as their primary language (Rodriguez et al., 2018). The reviewed publications provide only indirect insight into dairy farm employees' work-related values and beliefs because the studies were not designed to collect these data in a representative manner. Employees consistently demonstrated interest in continuing education, even in areas beyond their immediate duties, such as farm management and artificial insemination (Rovai et al., 2016; Alanis et al., 2022). However, owners and managers often underestimate the importance of regular training (Durst et al., 2018; Heuwieser et al., 2024). Confidence gains were often reported as results of training. For example, after e-learning on milking equipment, 95% and 86% of milkers reported feeling capable of inspecting the equipment and reporting issues to management, respectively (Alanis et al., 2022). Similarly, interactive euthanasia training increased participants' confidence in identifying compromised animals and making euthanasia decisions (Merenda et al., 2023) or correctly performing certain calf care tasks (Neukirchner et al., 2024). Most employees (81% to 94%) considered consistent task performance and protocol adherence important, and SOP-based training further increased their confidence in performing tasks reliably (Neukirchner et al., 2026). Formal recognition of training was highly appreciated, with 95% of workers viewing certificates as valuable for both current and future jobs (Rovai et al., 2016). Additionally, employees expressed a strong desire for more managerial involvement during training sessions (Rovai et al., 2016) and more frequent feedback or honest communication (Moore et al., 2020), indicating that visible engagement from owners and managers is important. Finally, many caretakers viewed situations such as euthanasia (Merenda et al., 2023) or recognizing a sick calf (Neukirchner et al., 2026) not only as a humane necessity but also as a personal failure to provide proper care.

The 15 manuscripts reviewed explore the development, delivery, and evaluation of training programs aimed at dairy farmworkers in various settings and with different methodologies in the United States and Germany. A common theme is the practical and logistical challenge of training a workforce characterized by high turnover, limited formal education, and, in US dairy farms, a predominantly Latino demographic with varying literacy levels. Training methods include e-learning, online courses based on SOP, in-person active learning sessions, and antimicrobial stewardship programs. Outcomes measured include knowledge gain, behavioral change, procedural adherence, milk quality indicators, and safety practices. Most studies show meaningful improvements in worker knowledge and confidence, but fewer demonstrate lasting behavioral change (Heuwieser et al., 2024), measurable herd-level results (Rodriguez et al., 2025), or effects on safety (Rovai et al., 2016). Achieving meaningful outcomes requires training that is active, supportive, and culturally sensitive. Singular training events and passive distribution of materials or isolated knowledge gains are not enough to change behavior or reduce antimicrobial use without continued reinforcement or management support.

Across the 15 publications reviewed, several consistent methodological patterns emerge that have direct implications for the rigor and generalizability of dairy worker training research. Only 1 study (Meyerhoff et al., 2016) reported conducting a training needs analysis before intervention design, even though training programs may be less effective when their content is not informed by a specific needs assessment, regardless of the delivery format (Norman et al., 2004). Although some studies conducted informal pre-intervention farm assessments such as equipment assessments or parlor evaluations (Alanis et al., 2022; Rodriguez et al., 2025) or literature-based gap identification (Garzon et al., 2023) that partially informed content development, none employed a structured process to identify learning needs (“pain points”) before training implementation. This finding suggests that training programs in the dairy industry are often developed without systematically assessing workers' learning needs, which may reduce their effectiveness (Norman et al., 2004). None of the reviewed publications reported having information on employees' core values, goals, fears, concerns, work conditions, or leisure activities. Understanding these aspects could help develop the workforce through better-aligned training strategies, types (onboarding vs. refresher) and storytelling techniques that are more engaging for the target audience and support better outcomes for both trainees and farms. This information may be particularly important when developing online modules for diverse audiences, because participants' knowledge and skill levels are often unknown until feedback is obtained after course completion. However, such learner-centered training design remains constrained by the limited qualitative research on dairy employees (Ritter et al., 2023).

Training efforts were also constrained by limited organizational support, including the absence of a strong learning culture, designated training spaces, or dedicated safety personnel. Production demands often made it difficult to release workers for training (Heuwieser et al., 2024; Rodriguez et al., 2025). High workforce mobility further affected program implementation and evaluation. In one milking routine study, the median time in position was only 9 mo, and 70% of participants had <1 yr of tenure (Rodriguez et al., 2025). In another study, 40% of milkers had worked on their current farm for <6 mo (Alanis et al., 2022). Additional limitations included high annual employee turnover, with a median turnover rate of 33% (Rodriguez et al., 2025), and limited employee availability for repeated training events or assessments, resulting in 17% to 20% attrition (Heuwieser et al., 2024). Limited internet connectivity and lack of device ownership were not discussed as potential barriers. However, in studies that provided implementation details, employees accessed training using tablets provided by the investigators during paid work time (Alanis et al., 2022; Heuwieser et al., 2024).

Sample size calculations were reported in 2 studies to detect significant changes in knowledge scores (Merenda et al., 2023; Rodriguez et al., 2025), but not for changes in employees' behavior or farm performance indices. None of the studies used random sampling; all relied on convenience sampling or voluntary recruitment. This approach is appropriate because sampling frames for dairy farm employees are generally unavailable, and human-subjects research typically requires informed consent. We therefore consider the sampling approach a limitation of external validity, particularly for generalizing findings to the broader dairy industry.

Ten studies used pre- and post-test comparisons, 4 used a post-test-only design, and 1 compared a hands-on approach with online delivery. Only 2 studies conducted follow-up assessments beyond the immediate post-training period, at 2 to 3 wk (Heuwieser et al., 2024) and 3 to 6 mo (Rodriguez et al., 2018) after training. All other training studies limited evaluation to immediate pre- and post-assessments. Several authors acknowledged this gap and called for future research on long-term knowledge retention, behavioral drift, and sustained program impact. The absence of delayed assessments leaves the durability of training effects largely unknown across this body of literature. Furthermore, in most studies that reported knowledge gains after a training intervention, the number of test items ranged from 3 to 10, which is lower than the median of 20 test items suggested in a recent meta-analysis (Edelsbrunner et al., 2025) for rigorous training experiments.

Evidence-based learning models have been developed to guide adult education in workplaces. For deskless workers, such as dairy employees, who often face time constraints, limited formal schooling, and language barriers, training should adhere to established principles of adult learning. Most studies were not grounded in an explicitly named learning model or educational theory. Only 3 studies applied named frameworks to justify training design or evaluation structure; most interventions relied on implicit instructional approaches. Blended learning, defined as a mix of in-person and online instruction, was uncommon and used in only 2 studies (Garzon et al., 2023; Portillo-González et al., 2024). Most studies used either online-only or in-person-only formats. Literacy was addressed with varying levels of rigor in approximately half of the studies. Approximately half of the studies addressed literacy, most commonly among Spanish-speaking immigrant workers, for whom low literacy was identified as a communication barrier. We recommend assessing literacy during the pre-training needs assessment to inform training design rather than as part of the training evaluation.

Taken together, these patterns suggest that research on dairy worker training lacks consistent application of instructional design principles, theoretical learning frameworks, and longitudinal evaluation methods.

Training design should be explicitly grounded in established learning theories, and blended delivery formats should be explored more systematically. Participant literacy and learning preferences should be formally assessed and reported as a standard demographic variable. To transform promising but inconsistent outcomes into reliable behavior change and herd-level improvements in key performance indicators, the next step is to design training grounded in clear adult learning principles that guide goal-setting, hands-on practice, feedback, assessment, and spaced repetition.
